# A novel lytic polysaccharide monooxygenase from enrichment microbiota and its application for shrimp shell powder biodegradation

**DOI:** 10.3389/fmicb.2023.1097492

**Published:** 2023-03-15

**Authors:** Yang Zhang, Delong Pan, Peiyao Xiao, Qianqian Xu, Fan Geng, Xinyu Zhang, Xiuling Zhou, Hong Xu

**Affiliations:** ^1^School of Life Science, Liaocheng University, Liaocheng, Shandong, China; ^2^College of Food Science and Light Industry, Nanjing Tech University, Nanjing, China

**Keywords:** enrichment, lytic polysaccharide monooxygenase, chitin oligosaccharides, synergies, antioxidant activity

## Abstract

Lytic polysaccharide monooxygenases (LPMO) are expected to change the current status of chitin resource utilization. This study reports that targeted enrichment of the microbiota was performed with chitin by the selective gradient culture technique, and a novel LPMO (M2822) was identified from the enrichment microbiota metagenome. First, soil samples were screened based on soil bacterial species and chitinase biodiversity. Then gradient enrichment culture with different chitin concentrations was carried out. The efficiency of chitin powder degradation was increased by 10.67 times through enrichment, and chitin degradation species *Chitiniphilus* and *Chitinolyticbacter* were enriched significantly. A novel LPMO (M2822) was found in the metagenome of the enriched microbiota. Phylogenetic analysis showed that M2822 had a unique phylogenetic position in auxiliary activity (AA) 10 family. The analysis of enzymatic hydrolysate showed that M2822 had chitin activity. When M2822 synergized with commercial chitinase to degrade chitin, the yield of *N*-acetyl glycosamine was 83.6% higher than chitinase alone. The optimum temperature and pH for M2822 activity were 35°C and 6.0. The synergistic action of M2822 and chitin-degrading enzymes secreted by *Chitiniphilus* sp. LZ32 could efficiently hydrolyze shrimp shell powder. After 12 h of enzymatic hydrolysis, chitin oligosaccharides (COS) yield reached 4,724 μg/mL. To our knowledge, this work is the first study to mine chitin activity LPMO in the metagenome of enriched microbiota. The obtained M2822 showed application prospects in the efficient production of COS.

## 1. Introduction

Chitin is the second-most abundant biosynthetic bio-polysaccharide after cellulose and is found in large quantities in shrimp and crab seafood waste (Zhang et al., 2020). Most of these wastes are burned or dumped in coastal areas, causing environmental problems such as water pollution (Duhsaki et al., 2022). Chitin and its derivatives are widely used in biomedicine, cosmetic refinement, food, and agriculture. However, their high crystallinity and insolubility in water remarkably limit their efficacy (Zhang et al., 2016; Xie et al., 2021). α-chitin is the most abundant chitin, has antiparallel chains and strong hydrogen bonds, and is the most crystalline and difficult to handle (Mukherjee et al., 2020). Chitin oligosaccharides (COS) are soluble, possess excellent bioactivity, and are expensive and vital chitin products. The chemical production method is commonly used in COS production. Not only is the hydrolysis reaction difficult to control, but also a great challenge to environmental protection (Kumar et al., 2021). The enzymatic production of COS has high product specificity and mild reaction conditions and is the trend in industrialization. However, due to the high crystallinity of chitin, most chitinases require special substrates such as colloidal chitin (CC), solubilized chitin, and other noncrystalline chitin, which have been treated with acid or base, in order to realize large-scale COS enzymatic production. Recent studies have shown that the combination of chemical pretreatment and enzymatic method can increase the porosity of chitin and improve the efficacy of chitin hydrolase. Especially, ionic liquids such as Trihexyltetradecylphosphonium bis (2, 4, 4-trimethylpentyl) phosphinate can significantly increase the yield of COS, which is a green, sustainable and promising production method (Kumar et al., 2021). Therefore, industrial enzymes face a major challenge in terms of stability, efficiency, and cost of their application (Nakagawa et al., 2013; Liaqat and Eltem, 2018). Microorganisms degrade and/or modify chitin in nature by using a series of synergistically acting chitinolytic enzymes (Duhsaki et al., 2022). Mining novel, highly efficient chitin-degrading enzymes from natural environments will lay a solid foundation for the industrial production of COS.

LPMOs can oxidatively cleave glycosidic bonds in difficult to degrade biological substances such as cellulose or chitin, thus depolymerizing these carbohydrate polymers (Vandhana et al., 2022). More and more LPMOs discovery has revolutionized the enzymatic conversion of recalcitrant polysaccharides (Mekasha et al., 2020). The pre-LPMOs times began in 1954. Previously, according to the similarity of amino acid sequence and active function, LPMOs were divided into two categories: carbohydrate binding module 33 family (CBM33) and glycoside hydrolase 61 family (GH61). Until 2010, Vaaje-Kolstad et al. (2010) demonstrated that a 21 kDa chitin binding protein (CBP21) could cleavage by redox reactions β-glycosidic bonds in chitin, it is marked a rotating point in the field of glycan processing enzymes. This discovery stimulated a lot of research work. In 2012, GH61 and CBM33 enzymes were uniformly classified as “LPMO.” LPMOs have a highly conserved planar structure similar to the β-sandwich folding of IgG (immunoglobulin G) and histidine scaffold. β-Sandwich fold consisting of 8–10 antiparallel β-Fold pass α-Helices are interconnected, and the histidine scaffold consists of an N-terminal histidine and an internal histidine with two histidines coordinating the Cu (II) ion through three nitrogen atoms to form the copper ion active center (Vaaje-Kolstad et al., 2017). In addition, conserved tyrosine (the phenylalanine in AA10 and AA15) is also necessary for catalysis (Gregory et al., 2016). LPMOs were previously thought to use molecular oxygen as a cosubstrate. However, Bissaro et al. (2017) research shows that these enzymes prefer hydrogen peroxide, so LPMOs work as peroxygenases rather than strictly monooxygenases. LPMOs are usually enzymes with multiple domains, and a significant portion of LPMOs have carbohydrate binding modules (CBMs) in addition to the catalytic domain. The presence of CBM not only promotes enzyme substrate binding and affects the substrate spectrum of LPMOs and may be involved in H_2_O_2_ production from LPMOs (Stepnov et al., 2022).

With the development of research, the substrates of LPMOs have expanded from chitin and cellulose to xylan, starch or pectin. LPMOs are distributed in many organisms, including bacteria, fungi, viruses, plants and insects. Currently, in the CAZy database, LPMOs are assigned to eight AA families (AA9-AA11 and AA13-AA17) of the helper activity (AA) family. Each family has a different biological origin and substrate specificity (Vandhana et al., 2022). LPMOs provide more binding sites for Glycoside Hylases (GHs) through oxidative cleavage of refractory polysaccharide crystalline structure, thus promoting the degradation of substrates by the GHs system. LPMOs combined with GHs can significantly improve the conversion efficiency of refractory polysaccharides, which has caused more and more basic and applied research in the field of bio-refining (Tamburrini et al., 2021).

As natural biomass, shrimp and crab shells can be completely degraded by microorganisms in their natural environment. This is accomplished by the synergistic action of chitin-degrading enzymes produced by microorganisms (Wieczorek et al., 2014). Many strains and enzymes with chitin-degrading activity have been screened and show remarkable potential in converting chitin into high value-added derivatives and pest control (Berini et al., 2019; Manjeet et al., 2019; Peng et al., 2020; Duhsaki et al., 2022; Wang et al., 2022). The synergistic effect of LPMO and chitinase on crystalline chitin has attracted the great attention of researchers at home and abroad. Many LPMOs have shown attractive application prospects in improving the efficiency of chitinase enzymatic hydrolysis. For example, four different LPMOs derived from *Streptomyces griseus* show the catalytic facilitation of several chitinases (Nakagawa et al., 2020). Chitinase and LPMO from *Aeromonas salmonicida* could increase the degradation efficiency of chitin powder by more than two times (Pentekhina et al., 2020). LPMO from *Trichoderma guizhouense* and chitinase increased the α-chitin and β-chitin degradation efficiency by 39.9 and 288.2%, respectively (Ma et al., 2021). A novel LPMO from *Bacillus amyloliquefaciens*, which synergized with glycoside hydrolase to degrade microcrystalline cellulose and colloidal chitin, the reducing sugar content increased by 7 and 23% (Guo et al., 2022). At present, the study of chitin-active LPMO is far less than that of cellulose-active LPMO. Therefore, novel chitin-active LPMO acquisition, studies of enzymatic properties, enzyme modification, and collaborative multi-enzyme degradation systems are now emerging as hot directions in chitin resource development (Mekasha et al., 2017; Hamre et al., 2019).

The enrichment culture is a common and effective method to increase the biomass of targeted microorganisms. Selective enrichment culture techniques have been widely used to enrich microbial degradation microbiota of contaminants and biomass to effectively obtain the efficient degradation microbiota or strains of the corresponding substrates (Park and Oh, 2020; Zhang J. et al., 2020; Wang et al., 2021). Accordingly, the study on the enrichment of chitin-degrading microbiota is of great significance to mining novel chitin-degrading enzymes. However, the research information is very limited. In this study, the targeted enrichment of chitin-degrading microbiota is performed by the selective gradient enrichment technique for the first time. Based on high-throughput sequencing analysis of the macrogenome, the novel LPMO M2822 is investigated. The enzymatic properties and chitin degradation performance of M2822 are studied. To the best of our knowledge, LPMO is obtained in the enrichment macrogenome for the first time, and its properties and applications are investigated. These results extend the application of the gradient culture technique and indicate the potential application of M2822 in the biorefinement of chitin-rich waste.

## 2. Materials and methods

### 2.1. Strains, plasmids, and chemicals

*Escherichia coli* DH5α and *E. coli* BL21(DE3) were used in the sub-cloning and expression of the LPMO gene from the metagenome, respectively. The pET-22b(+) plasmids were used as the expression vector of the LPMO gene. The chitin oligosaccharides (polymerization degrees 2 to 6) were acquired from Qingdao HEHAI Biotech Co., Ltd. (Qingdao, China).

The 2,6-Dimethoxyphenol (2,6-DMP), chitinase and chitin powder (CP) were purchased from Shanghai Yuanye Bio-Technology Co., Ltd. (Shanghai, China). The shrimp were purchased from a local seafood market to obtain shrimp shells, washed thoroughly with tap water to remove residual shrimp meat, and dried in a vacuum oven at 60°C for 24 h. The dried material was pulverized in a disintegrator and then passed through a 40 mesh sieve to produce shrimp shell powder (SSP). Preparation of β-chitin according to the Ma et al. (2021) method. Colloidal chitin (CC) was prepared following a previous method (Zhang et al., 2017). The remaining reagents were of analytical grade and used without additional purification.

### 2.2. Gradient enrichment of chitin degrading bacterial community

Soil samples were collected in Liaocheng, Shandong, China (116°0167′N,36°4,336′E). 1 g soil sample was inoculated into a 250 mL flask containing 50 mL enrichment medium and incubated at 30°C and 180 rpm. The culture was finished when the CP in the fermentation broth was degraded to be invisible to the naked eye. The next batch of enrichment culture was carried out with 15% inoculation until the degradation of CP could be completed within 3 days. The enrichment medium comprised of 2 g/L peptone, 1 g/L glucose, 0.7 g/L K_2_HPO_4_, 0.3 g/L KH_2_PO_4_ and 0.5 g/L MgSO_4_, and chitin. The gradient acclimation was carried out with chitin content of 1 g/L, 2 g/L, and 4 g/L.

### 2.3. High-throughput sequencing and bioinformatics analysis

#### 2.3.1. Microbial diversity sequencing and analysis of soil samples

The total DNA of soil samples was extracted by E.Z.N.A. soil DNA kit (Omega Bio-Tek, Norcross, GA, U.S.). After quality inspection and quantification, the V3-V4 region of 16S rRNA was amplified by PCR using 338F (5´-ACTCCTACGGGAGGCAGCAG-3′) and 806R (5´-GGACTACHVGGGTWTCTAAT-3′). The conserved region of chitinase was amplified by combining primers ChiF (5´-CGTGGACATCGACTGGGARTWYCC-3′) and ChiR (5´-CCCAGGCGCCGTAGARRTCRTARSWCA-3′). PCR products were detected, quantified and sequenced by the Miseq PE300 platform of Illumina Company. The fastp software was used for quality control of original sequencing sequences, and Flash software was used for splicing. Using Usearch software,^1^ the sequences were clustered by OTU according to 97% similarity, and chimerism was eliminated. RDP classifier^2^ was used to compare each sequence with the Silva database, the comparison threshold was set to 70%, and the annotation results of species classification were obtained.

#### 2.3.2. Metagenomic sequencing, analysis and CAZyme annotation

The entire DNA of the enrichment bacterial community was extracted using the E.Z.N.A. soil DNA kit. After concentration, purity and integrity testing, ultrasound treatment was performed, and a 450 bp insertion library was constructed. The fastp software is used to filter the original data, megahit is used to mix and splice multiple samples, bowite2 is used to extract unmapped reads, and SPAdes is used to splice again to get more complete contigs assembly results. Prodigal was used to predict the ORF of splicing results, and CD-HIT software was used to obtain non-redundant gene sets. Phylogenetic trees were drawn with GraPhlAn (Asnicar et al., 2015). Use the HMMER algorithm for the Carbohydrate-Active enZYmes Database (CAZy) comparative analysis on the dbCAN2 network server (Zhang et al., 2018).

#### 2.3.3. Bioinformatics analysis of M2822

InterProScan was used to search and analyze conserved domains and feature sequences. Signal peptide analysis was performed with signal P5.0 server. The map of the protein domain was drawn by IBS 1.0.3 software. Phylogenetic analysis was performed using MEGA7 software and optimized and annotated on the iTOL web server. ClustalX software was used for sequence alignment between full-length M2822 and five characteristic LPMO10s. Sequence similarity and secondary structure analyses are carried out using ESPRIPT 3.0 network server. Modelling structural homology in dense modelling mode using a Phyre2 server (Kelley et al., 2016). The model was observed and annotated using Visual Molecular Dynamics (version 1.9. 4a53) (Humphrey et al., 1996).

### 2.4. Cloning, expression, and purification of M2822

Because M2822 contains a secretion signal peptide, the first 28 amino acids are eliminated to avoid their potential influence on the solubility of the protein. The M2822 DNA fragment was synthesized with codon optimization by GENEWIZ Inc. (Suzhou, China). The PCR fragment was inserted between pelB and His pET-22b(+) vector tag to construct recombinant plasmid pET-22b-m2822. The recombinant plasmid was transformed into *E. coli* BL21 (DE3) to construct M2822 expression strain *E. coli* BL21-M2822. It was inoculated into a new LB medium containing ampicillin (50 μ g/ml) and cultured at 37°C at 200 rpm until the OD_600_ nm value was 0.6. The culture was cooled to 15°C and left standing for 30 min. Isopropyl β-d-1-thiogalactopyranoside (IPTG) was added to the final concentration of 0.5 mmol/l, cultured at 15°C for 8 h, centrifuged at 8000 rpm for 10 min to collect the cells. Extraction of M2822 protein from the periplasm of *E. coli* BL21-M2822 using an osmotic shock method (Humphrey et al., 1996). The soluble M2822 protein was purified by nickel-chelating chromatography, then concentrated and desalted by 10 kDa ultrafiltration tube. Copper saturation of purified proteins was performed using the method described by Loose et al. (2014). Excess copper was removed using a Sephadex desalting column. The Bradford method determined the concentration of M2822 protein after desalting and concentrated by an ultrafiltration tube as needed.

### 2.5. M2822 activity assay

The chitin degradation activity of M2822 was carried out according to our previous study and made minor modifications (Zhang et al., 2017). The reaction system was Britton-Robinson buffer (pH 6.8), 2.5 μM M2822, 10 mg/ml CP and 1 mM ascorbic acid, incubated at 37°C for 24 h, and then heated at 100°C for 10 min to terminate the enzyme reaction. The Britton-Robinson buffer was prepared with 0.04 mol/L phosphoric, boric, and acetic acid. When used, 0.2 mol/L NaOH solution was raised to the required pH value in the acidity meter. The enzymatic hydrolysate was purified by SPE column containing graphite carbon and then analyzed by MALDI-TOF MS. Simply, samples were analyzed in positive collection mode using UltrafleXtreme MALDI-TOF/TOF (Bruker Daltonics GmbH, Bremen, Germany) with 2, 5-DHB matrix. The acquisition was performed in reflectron mode with an accelerating voltage of 20 kV and a reflected layer voltage of 21.1 kV in the acquisition range from m/z 400 to 3,000.

### 2.6. M2822 substrate binding detection

Microcrystalline cellulose (MCC), powdered α-chitin, powdered β-chitin and colloidal α-chitin (CC) were added into M2822 solution at a concentration of 10 g/L, respectively, incubated at 25 ° C and 200 rpm for 30 min, then centrifuged at 6000 rpm for 10 min to collect the supernatant. The protein content of the solution before substrate addition and the supernatant after incubation was examined using the BCA protein assay kit. The binding ability of M2822 to the insoluble substrate was evaluated by the decrease of M2822 in the supernatant.

### 2.7. Synergy of chitin degradation between M2822 and commercial chitinase

A synergistic reaction system of M2822 and commercial chitinase was established in Britton-Robinson buffer (pH 6.8) using powder α-chitin as substrate. The reaction system contained 10 mg/ml chitin, 1 mg/mL chitinase, 100 μ mol/L H_2_O_2_ and 0.143 mg/mL M2822. The samples were incubated at 30°C under 300 rpm shaking for 12 h, and samples were taken to detect at selected time points. The content of *N*-acetyl glucosamine (GlcNAc) produced by enzymatic hydrolysis was quantitatively detected according to the HPLC detection method of Madhuprakash et al. (2015). The 10 μL reaction mixture was resolved on an Alltima HP HILIC column (4.6 ID × 250 mm) by isocratic elution with 80% acetonitrile at a 1 mL/min flow rate, and the absorbance was monitored at 195 nm.

### 2.8. Enzymatic properties of M2822

The activity of M2822 was determined with 2, 6-dimethylphenol (2, 6-DMP) and H_2_O_2_ as substrate according to the method of Breslmayr et al. (2018), with some modifications. Because of the existence of 2, 6-DMP and H_2_O_2_ substrates in the reaction system. The additional amounts of 2, 6-DMP and H_2_O_2_ in the reaction system were determined by optimization. The 200 μL reaction system contains 2.5 μM M2822, 100 μm H_2_O_2_ and different concentrations of 2, 6-DMP (1–50 mmol/L). After incubation at 30°C for 15 min, the M2822 was added to the enzyme-labelled plate, and then the reaction was carried out for 300 s at 30°C. The absorbance value increment of the reaction product coerulignone at 469_nm_ was measured. The amount of enzyme needed to produce 1 μM coerulignone in 1 min was defined as one enzyme activity unit (U). Under the condition of 5 mmol/L 2, 6-DMP, the optimal conditions were obtained by changing the concentration of H_2_O_2_ (10–500 μ mol/L).

In order to investigate the temperature effect on M2822 activity, the enzyme reaction was performed with 5 mM 2,6-DMP and 0.1 mM H_2_O_2_ as substrates in Britton-Robinson buffer at pH 6.8 in the temperature range of 25–50°C. The highest enzyme activity determined is defined as relative 100%. To evaluate the thermal stability of M2822, it was treated at different temperatures (30, 35, 40, 50°C) for 8 h, and samples were taken at selected time points to detect the residual activity of M2822. The initial activity of m2822 was 100% in each condition. According to Madhuprakash et al. (2015) method, the optimum pH of M2822 was determined by using glycine-hydrochloric acid (pH 2.2–3), sodium citrate (pH 3–6), sodium phosphate (pH 6–8), trihydrochloric acid (pH 7.2–9) and glycine-sodium hydroxide (pH 9–10) buffers in the pH range of 2.2–10. The Britton-Robinson buffer was adjusted to different values of pH 3–11 and also used to study the effect of pHs on M2822 activity. The highest measured M2822 activity was standardized to 100%. In order to evaluate the pH stability of the M2822 protein, it was incubated in the Britton-Robinson buffer with different pH values for 1 and 10 h. The initial M2822 activity without incubation in different buffers was considered as 100%.

The kinetic parameters of M2822 for H_2_O_2_ were determined under the optimum conditions. 2.5 μM M2822, 5 mmol/L 2, 6-DMP and different concentrations of H_2_O_2_ (5–500 μmol/L) were incubated at 35°C for 300 s in the Britton-Robinson reaction system at pH 6.0.

### 2.9. Enzymatic hydrolysis of chitin by M2822 in cooperation with *Chitiniphilus* sp. LZ32 secretase

Crude enzyme solution of *C.* sp. LZ32 (ChiMix32) was prepared according to the previous method (Zhang et al., 2017). M2822 (25 μ mol/L) and ChiMix32 (1.5 mg/ml) were mixed to prepare chitin-degrading enzyme cocktails. The synergistic degradation experiments of colloidal chitin, chitin powder (CP, 100 mesh) and shrimp shell powder (SSP, 100 mesh) were carried out by enzyme cocktails. Because ChiMix32 has the highest COS yield in the Britton-Robinson reaction system at pH 6.5 when chitin is used as substrate (data not shown). Therefore, the synergistic reaction system of M2822 and ChiMix32 is a Britton-Robinson buffer with pH 6.5 10 mg/ml chitin, 100 μmol/L H_2_O_2_. After adding 10% enzyme cocktails, the enzyme hydrolysis was carried out at 30°C and 200 rpm for 12 h, and samples were taken at the selected time point. The content of different degrees of COS was quantitatively detected following the HPLC detection method of Zhang et al. (2017).

## 3. Results and discussion

### 3.1. Enrichment of chitin-degrading microbiota

**Figure 1:**
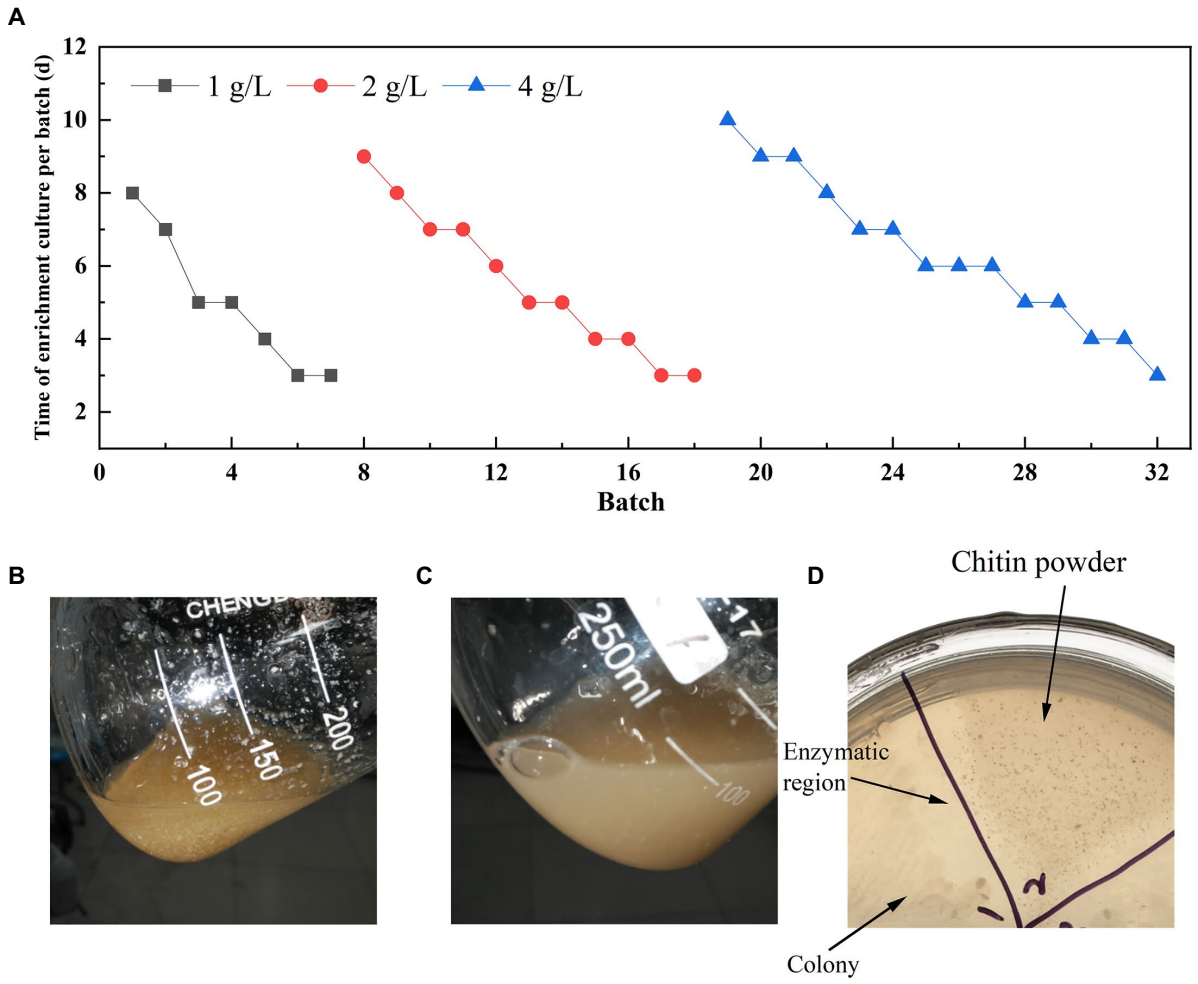
Enrichment results of LNM in different concentrations of chitin powder. **(A)** Time for gradient enrichment in each batch; Appearance state of chitin powder in the fermentation broth of LNM32 before inoculation **(B)** and after 3 days **(C)** of fermentation; **(D)** LNM32 hydrolyzed chitin powder produced clear hydrolyzed area on the agar plate.

Soil samples were analyzed for diversity through 16 s rRNA universal primers (338F and 806R) and glycoside hydrolase family 18 chitinase degenerate primers (ChiF and ChiR) (Xu et al., 2016). Three soil samples were selected for the enrichment of chitin degradation based on the analysis results. Finally, one sample (named LNM) was enrichment for 32 batches to obtain an enrichment microbiota (LNM32) with efficient degradation viability of powdered chitin. The duration of a single batch was increased significantly with increasing concentration of powdered chitin during enrichment (Figure 1A). After enrichment under three concentration gradients of 1, 2, and 4 g/l, LNM32 could completely degrade 4 g/l chitin powder (CP) in 3 days (Figures 1B,C). LNM32 was inoculated onto agar plates with CP as substrate. After culture, the CP around the colony dissolved and disappeared, forming a dissolution circle visible to the naked eye (Figure 1D). The LNM32 degradation efficiency on CP was 10.67 times that before enrichment, which showed that the gradient enrichment of soil microbiota by CP could effectively improve the degradation ability of the CP. LNM32 was an excellent material for studying chitin hydrolysis of powder particles with high crystallinity.

### 3.2. High-throughput sequencing analysis of enrichment microbiota

The macro genome of LNM32 generated 1.597 Gb of original sequence data. After data quality control, clean reads splicing and assembly, and gene prediction, a non-redundant gene set was constructed. The number of genes was 3,643, and the N50 value was 1,203 bp. The abundance of each gene was calculated and counted. The high-throughput sequencing of the LNM32 metagenome showed that all species in LNM32 were bacteria, of which phylum Proteobacteria, Class Betaproteobacteria, Order Neisseriales, Family *Chromobacteriaceae*, Family *Neisseriaceae,* Genus *Chitiniphilus*, and Genus *Chitinolyticbacter* accounted for 99.49, 96.35, 90.40, 61.10, 29.07, 49.70, and 28.61%, respectively.

**Figure 2:**
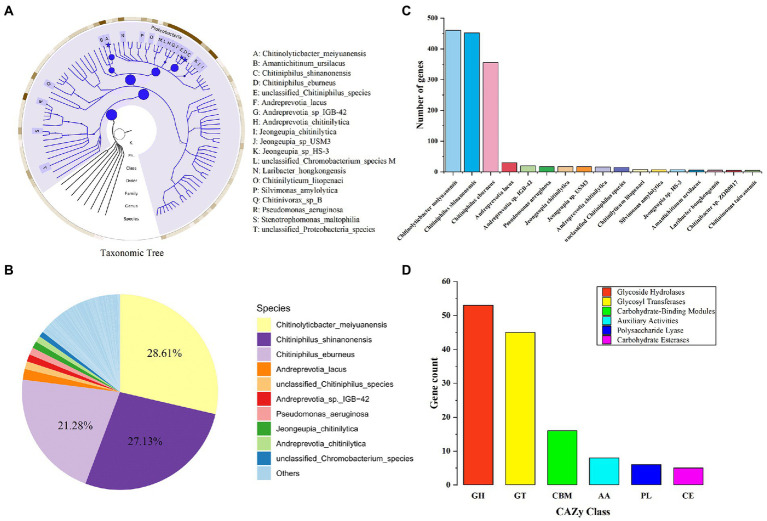
Metagenomics analysis of LNM32. **(A)** Phylogenetic tree of the microbial metagenome-assembled genome from the LNM32. The size of the circle and asterisk represents the abundance size; **(B)** Relative abundance at the bacterial species level; **(C)** The number of genes annotated to different species (shown in top 90%); **(D)**The number of genes annotated as each CAZy family.

Species taxonomic hierarchy annotation results showed that all species in LNM32 were bacterial, among which phylum Proteobacteria, Class Betaproteobacteria, and Order Neisseriales account for 99.49, 96.35, and 90.40% of their taxonomic hierarchy, respectively (Figure 2A). Under Order Neisseriales, the abundance of Family *Chromobacteriaceae* and Family *Neisseriaceae* is 61.10 and 29.07%, respectively (Figure 2A). At the species level, the three species with the highest abundance are *Chitinolyticbacter meiyuanensis*, *Chitiniphilus shinanonensis,* and *Chitiniphilus eburneu* and their abundance values are 28.61, 27.1 and 21.28%, respectively (Figure 2B). The number of genes annotated belonging to the three bacterial species was 457, 450, and 354, respectively (Figure 2C). Some studies showed that these three species have excellent chitin-degrading activity (Rani et al., 2020; Sheng et al., 2020; Zhang et al., 2020). The CAZy annotation results showed that LNM32 contained a rich and diverse carbohydrate-active enzyme (Supplementary Table S1). Further analysis showed that LNM32 harboured 115 CAZyme genes, including 53 glycosyl hydrolase, 45 glycoside transferases, 16 carbohydrate-binding modules, 8 auxiliary activities (AA), 12 polysaccharide lyases and 5 carbohydrate esterases (Figure 2D). Given the excellent ability of LNM32 to degrade powdered chitin, the AA10 family proteins contained in LNM32 were analyzed in detail. Through analysis, it is found that the M2822 protein annotated as AA10 family LPMO has high gene abundance (1,316 reads, accounting for 0.0192% of the total metagenome) and complete gene sequence. Zhang et al. (2020) analyzed the genome of *C. meiyuanensis*, it was found that there was only one AA10 family LPMO. The whole genomes of *C. burneus* (GCA_005048205. 1) and *C. shinanonensis* DSM 23277 (GCA_000374805. 1) were analyzed by dbCAN. The results showed that both strains had only one AA10 family LPMO. Therefore, AA10 family LPMO may play an essential role in the degradation of chitin by LNM32. M2822 was selected for further study.

### 3.3. Phylogenetic analysis, sequence comparative analysis, and structural simulation of M2822

**Figure 3:**
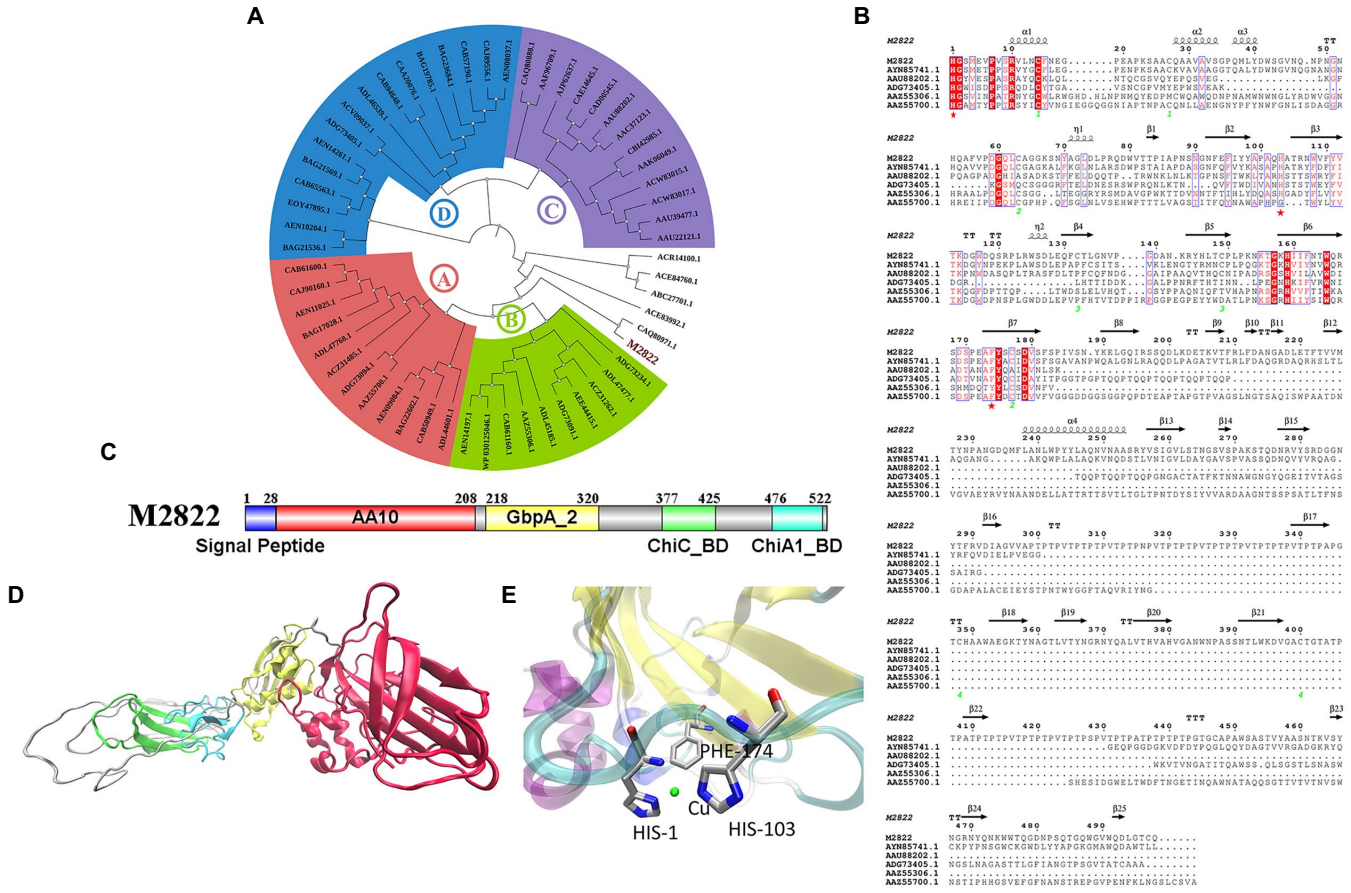
Bioinformatics analysis of M2822. **(A)** Phylogenetic tree analysis of M2822; **(B)** Multiple alignments of amino acid sequences of AYN85741.1, AAU88202.1, ADG73405.1, AAZ55306.1, AAZ55700.1 and M2822; **(C)** M2822 conserved domain analysis; **(D)** Cartoon representation of three-dimensional homology models of M2822. **(E)** Predicted 3-D model structures of M2822 active center.

The amino acid sequence of M2822 (Supplementary Table S2) was compared with the sequences in GenBank. Which had the best BLAST hit (99% query cover and 71.88% identity) with an LPMO from *C. shinanonensis* (BDB32680.1), and also showed 56.72% identity (99% query cover) to a LPMO from *C. piscinae* (WP_194115059.1). These LPMOs have not been further studied. A phylogenetic analysis of M2822 and previously reported typical AA10s (Nakagawa et al., 2015) showed that AA10s formed two major Clades, each containing two subClads. Clade, composed of Clads A and B, was primarily derived from actinomycetes and had chitin and cellulose activities (Figure 3A). Clade, composed of Clads C and D, mainly consisted of members with chitin activity (Deng et al., 2019). M2822 was not classified into four Clads, which converged in a single clade with Clads A and B. This finding might indicate that M2822 belonged to new unspecified Clads. Notably, CAQ80971.1, closely related to M2822, was recently confirmed to be LPMO with chitin activity (Skåne et al., 2021). The comparative analysis of M2822 with four Clade typical sequences showed that M2822 had a typical LPMO catalytic activity center. M2822 have two histidine residues bound to copper and an aromatic phenylalanine residue (e.g., His1, His103, and PHE174). In addition, alanine (Ala113), which contributed to the shaping of copper-binding sites (Hemsworth et al., 2013), was found in the vicinity of His103 (Figure 3B).

Sequence analysis showed that M2822 had 526 amino acid residues. The first 28 amino acids were predicted to be signal peptides (Figure 3C). Currently, increasing LPMOs are found to have multiple conserved domains, which play an essential role in the substrate binding and catalytic efficiency of LPMOs (Courtade and Aachmann, 2019; Mekasha et al., 2020). In addition, some domains may also protect LPMOs from autocatalytic inactivation (Dade et al., 2022). The analysis of conserved domains showed that M2822 contained four conserved domains (Figure 3C). These domains are AA10 catalytic domain (amino acids 29–208), *N*-acetylglucosamine-binding protein domain 2 (amino acids 218–320), and chitin-binding domain of chitinase C (amino acids 377–425), and chitin-binding domain of Chi A1-like proteins (amino acids 476–522). Based on the function of each conserved domain, M2822 was predicted to be an LPMO with chitin activity in the AA10 family. The Phyre2 server (Kelley et al., 2016) was used to generate a three-level structure homologous model for M2822 by using C4nz3A, c7sqxA, C6z40A, 2d49A, and 2xwxB as templates (Figure 3D). The predicted M2822 structural model showed that M2822 had a typical fibronectin/immunoglobulin-like β-sandwich structure AA10 domain, which comprised two antiparallel β-sheets connected by loop regions. The Cu-binding site formed by His1, His103, and PHE174 was located in β-sheets between sandwich structures (Figure 3E). In addition, the model showed that M2822 had a GbpA2 domain, which had an *N*-acetylglucosamine-binding function, and chitin-binding domains of families 19 chitinase C and 18 chitinase A1 (Wong et al., 2012). This finding indicated that M2822 had a broad substrate spectrum and could degrade insoluble chitin. The above bioinformatics analyses suggested that M2822 might be a novel LPMO in AA10 with chitin activity.

### 3.4. M2822 expression and enzymatic hydrolysate analysis

**Figure 4:**
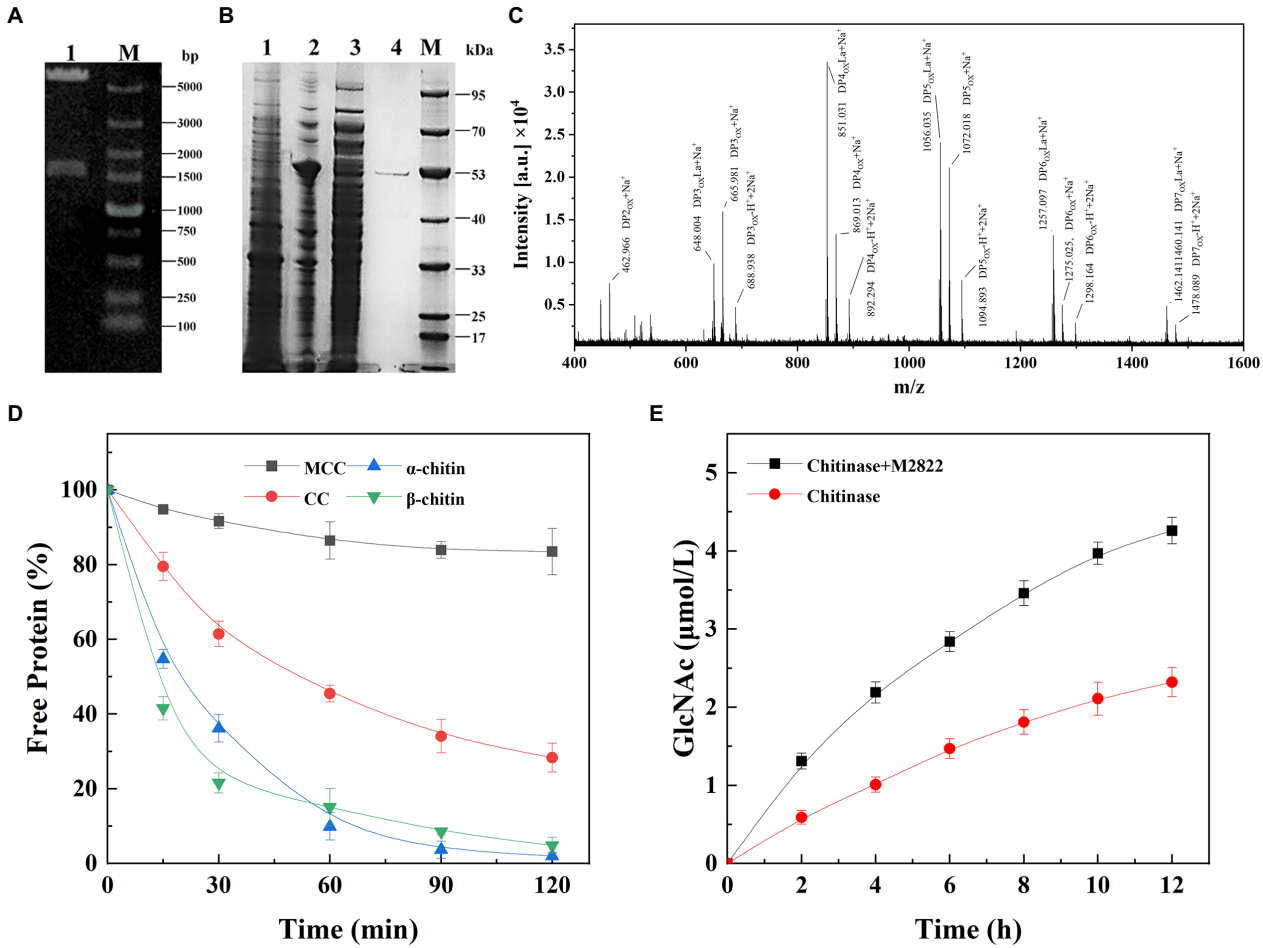
Expression, purification and activity assay of M2822. **(A)** Verification of M2822 gene expression plasmid by nucleic acid electrophoresis. Lane 1, pET-22b-m2822 XhoI and NdeI double enzyme digestion; lane M, DNA marker; **(B)** SDS-PAGE analysis of purification process of M2822. lane 1, contains no plasmid-expressed *E. coli* BL21 protein; lane 2, *E. coli* BL21-M2822 soluble protein; lane 3, *E. coli* BL21-M2822 global protein; lane 4, purified M2822 protein; Lane M, protein marker; **(C)** MALDI-TOF MS analysis of the products generated by M2822; **(D)** Adsorption capacity of different substrates to M2822; **(E)** Synergistic degradation of chitin by the M2822 with commercial chitinase.

The nucleotides of the M2822 gene were inserted into the pET-22b(+) vector (Figure 4A), and *E. coli* Bl 21 (DE3) was transformed and induced to be expressed by IPTG. The M2822 protein (54 kDa) was obtained by osmotic impingement method (Manoil and Beckwith, 1986), and purification by Ni column affinity chromatography (Figure 4B). Enzymatic hydrolysis was carried out with CP as substrate. The product was purified and analyzed by MALDI-TOF MS. The mass spectrometry analysis of the products generated from α-chitin showed that these were C1-oxidized COS with a main degree of polymerization 2 to 7. Mass spectra show that these C1-oxidized oligomers have characteristic clusters of signals corresponding to the Na^+^ salt of aldose acid (Figure 4C). For example, *m*/*z* 648.004, 851.031, 1056.035, 1257.097 and 1460.141 correspond to Na^+^ adducts of lactone forms with the degree of polymerization 3 to 7, respectively. *m*/*z* 665.981, 869.513, 1072.018, 1275.025 and 1478.089 correspond to the single Na^+^ salt of polymerization degrees (PD) 2 to 7 aldehyde acid. Similar results were reported in other studies on LPMOs (Li et al., 2022; Zhou et al., 2022). Therefore, M2822 was an LPMO with chitin C1 oxidative activity.

### 3.5. Substrate specificity of M2822

Purified M2822 was incubated with excess microcrystalline cellulose (MCC), α-CP, β-CP, and colloidal α-chitin (CC), and the content of free protein after the binding was detected to investigate the ability of M2822 to bind to insoluble substrates. As shown in Figure 4D, M2822 had the weakest binding ability to MCC. This further confirmed the function of the chitin-binding domain of chitinase (ChiC_BD and ChiA1_BD) contained in the M2822. The binding strengths of M2822 to the three other substrates were as follows: α-CP > β-CP > CC. M2822 was speculated to have two different chitin-binding domains, allowing strong binding onto crystalline chitin surfaces. For LPMOs, compared with enzymes containing only the catalytic AA10 domain, the presence of CBM enhanced LPMO binding to substrates and resulted in high yields of oxidation products (Forsberg et al., 2014; Courtade and Aachmann, 2019). The CBM prevented the unproductive reaction of reduced LPMO with its cosubstrate, which is the main contributor to enzyme inactivation (Forsberg et al., 2018; Mutahir et al., 2018). In addition, the adsorption of LPMO on chitin will help to achieve the reuse of chitinase, thereby improving the economy of enzyme utilization.

### 3.6. M2822 synergizes with chitinase to degrade chitin

The synergistic effect of M2822 on chitinase in the process of chitin degradation was verified by detecting the amount of GlcNAc produced in the reaction system. Synergistic experiments showed that the degradation of CP by chitinase was significantly improved in the presence of M2822 (Figure 4E). When M2822 combined with chitinase for 2 h, the yield of GlcNAc was 2.22 times that of chitinase alone. After 12 h of reaction, GlcNAc release concentration reached 4.26 μmol/mL, 83.6% higher than that produced by chitinase. Therefore, M2822 and commercial chitinase could degrade chitin cooperatively and improve the degradation efficiency effectively.

### 3.7. Enzymatic properties of M2822

**Figure 5:**
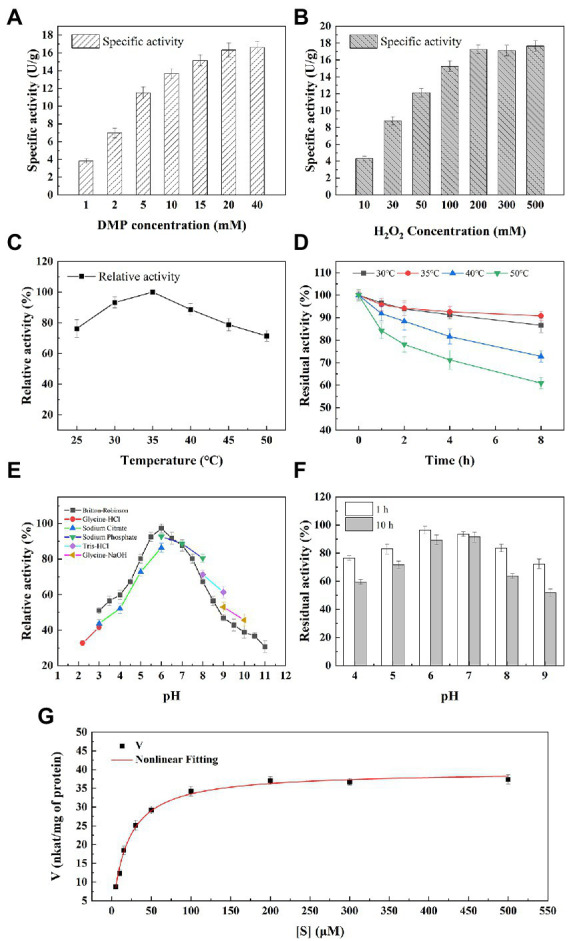
Enzymatic properties of M2822. Effect of 2,6-DMP **(A)** and H_2_O_2_ **(B)** concentration on M2822 activity; Influence of temperature on the activity **(C)** and stability **(D)** of M2822; Influence of pH on the activity **(E)** and stability **(F)** of M2822; **(G)** Michaelis–Menten kinetics were performed at optimal conditions.

The activity assay with 2,6-2,6-DMP as the colour substrate is a fast and sensitive activity assay for LPMOs (Breslmayr et al., 2018; Sagarika et al., 2022; Zhou et al., 2022). To find the best M2822 activity detection system, 2,6-DMP with different concentrations was used to examine the effect of 2,6-DMP on the activity determination of M2822 protein. Results showed that the specific activity of M2822 increased with increasing 2,6-DMP concentration, and the increase tended to be slow when the 2,6-DMP concentration was higher than 5 mM (Figure 5A). When the H_2_O_2_ concentration was increased under the condition of 5 mM 2,6-DMP, the specific activity that M2822 could detect will also be increased. When the H_2_O_2_ concentration was 200 μM, the specific activity detected was only slightly higher than 100 μM (Figure 5B), which was consistent with previously reported results by Breslmayr et al. (2018). Therefore, 5 mM 2,6-DMP and 100 μM were selected as conditions for M2822 enzyme activity detection.

The effect of temperature on the activity and stability of M2822 was investigated. As shown in Figure 5C, M2822 had good catalytic activity in the range of 20°C–50°C, and the oxidation activity of 2,6-DMP was highest at 35°C. In addition, the relative activity still exceeded 70% at 50°C. The thermal stability of M2822 was evaluated at 30°C, 35°C, 40°C, and 50°C. With prolonged incubation time, the M2822 activity decreased at different temperatures. After incubation at 30°C, 35°C and 40°C for 8 h, the residual activity of M2822 was 86.6, 90.8 and 72.8%, respectively. However, after incubation at 50°C for 8 h, M2822 only maintained the activity of 60.9% (Figure 5D). The optimum temperature for M2822 is 35°C, which has better thermal stability at this temperature.

The activity of M2822 at various pH values was examined in different buffer systems (Figure 5E). M2822 showed high activity in Britton-Robinson and Sodium phosphate buffer at pH 6.0, and the activity of Britton-Robinson was the highest. Britton-Robinson is more suitable as the buffer system of M2822. It should be noted that the activity values obtained by Britton-Robinson under alkaline conditions were lower than those obtained by the other three buffer systems. Therefore, the Britton-Robinson buffer system is unsuitable for the reaction of M2822 under alkaline conditions. In the Britton-Robinson buffer system, M2822 activity decreased significantly at pH 5.0 and pH > 7.5, but its relative activity still exceeded 50% at pH 3.0. The stability of M2822 was evaluated in the buffer solution with pH 4–9. Results are shown in Figure 5F. With the extension of incubation time, the activity of M2822 decreased especially under alkaline conditions. At pH 4.0 and 9.0, the residual enzyme activities after 10 h incubation were only 59.56 and 51.78%, respectively, of the initial value. M2822 showed the highest stability at pH 7.0, and the enzyme activity after 10 h incubation was only 2.13 percentage points lower than after 1 h. Therefore, M2822 had good adaptability to acidic conditions and had the best stability under neutral conditions.

Michaelis–Menten kinetic parameters for M2822 were determined with varied concentrations of H_2_O_2_. The curve fitting with non-linear regression function revealed the kinetic parameters Km and Vmax as 18.14 μM and 39.59 nkat/mg of protein (Figure 5G). The Km value of M2822 is lower than the reported Km value of NcLPMO9C from *Neurospora crassa* at pH 6.0 (Breslmayr et al., 2018). The low Km value of M2822 reflects its high affinity for substrates. In addition, the Kcat and total catalytic efficiency (Kcat/Km) of M2822 were 1.7 s^−1^ and 0.4 s^−1^ μm^−1^, respectively. The kinetic analysis under the optimum conditions showed that H_2_O_2_ was a suitable substrate for M2822, which promoted chitin degradation by M2822.

### 3.8. Shrimp shell chitin degradation by M2822 and chitin degrading enzymes

**Figure. 6:**
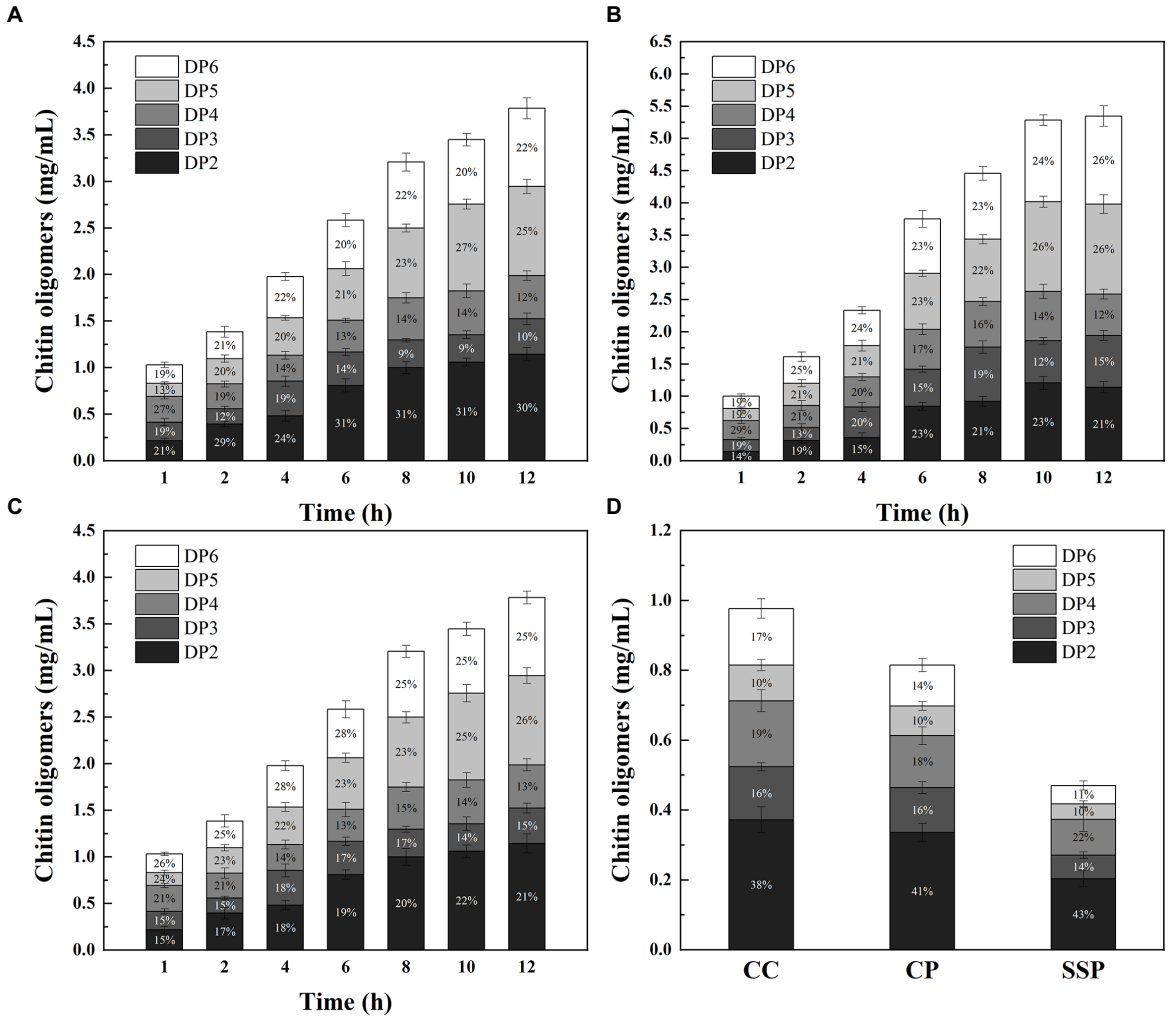
Time course of COS production from CC **(A)**, CP **(B)** and SSP **(C)** degradation by CDEC; **(D)** Time course of COS production from CC, CP and SSP degradation by ChiMix32.

To further evaluate the potential of M2822 in chitin degradation, chitin-degrading enzymes cocktails (CDEC) were prepared by combining M2822 with chitin-degrading enzymes. Considering the high cost of commercial chitin-degrading enzymes, the enzyme used to prepare CDEC is ChiMix32, which is a crude enzyme secreted by *C.* sp. LZ32. The *C.* Sp. LZ32 is a chitin-degrading bacterium that is isolated from soil. Our previous studies showed that *C.* sp. LZ32 has a good enzymolysis effect on chitin, which can hydrolyze the non-pretreated housefly larvae powder into a COS with a degree of polymerization of 2 to 6 (Zhang et al., 2017). Three substrates, colloidal chitin (CC), chitin powder (CP) and shrimp shell powder (SSP), were degraded by CDEC. The results showed that ChiMix32 and CDEC could produce COS with a polymerization degree of 2–6 after enzymatic hydrolysis of the three substrates (Figure 6). Compared with ChiMix32, CDEC produces more COS. After enzymatic hydrolysis for 12 h, the total amount of COS produced by CDEC using CC, CP and SSP as substrates was 3.79, 5.35 and 4.72 mg/mL, respectively. However, Chimix32 only produced 0.98, 0.82, and 0.47 mg/mL COS, respectively (Figure 6D). Although the total amount of COS produced by CDEC degradation of SSP was lower than that of CP but higher than that of CC, the possible reason is that M2822 is more adept at the degradation of crystalline chitin. In addition, the proportion of enzymatic hydrolysate with different polymerization degrees in COS is different. The proportion of PD5 and PD6 in the total production increased with the prolongation of enzymatic hydrolysis time. When CP and SSP were used as substrates, the amount of COS with high polymerization degree was higher than that of CC. After 12 h enzymatic hydrolysis of CC, CP and SSP, DP6 accounted for 22.1, 25.5 and 24.9% of the three enzymatic hydrolysates, respectively. When CC is used as a substrate, more COS with a low polymerization degree (DP2-4) can be produced. In summary, M2822 can promote the enzymatic hydrolysis efficiency of chitin-degrading enzyme, and it can produce more COS with a high polymerization degree with crystalline chitin as substrate.

## 4. Conclusion

This study obtained an enrichment microbiota with a high degradation performance of powdered chitin through the gradient enrichment of soil samples. A novel AA10 family LPMO M2822 was successfully captured. M2822 could cooperate with chitinase and significantly improve the degradation efficiency of powdered chitin. M2822 was expressed in *E. coli*, and its enzymatic properties were characterized. Enzyme cocktails composed of M2822 and chitin-degrading enzymes could degrade SSP into the COS of PD2-6. M2822 has significant application potential in the biorefinery of chitin-rich biomass.

## Data availability statement

The original contributions presented in the study are included in the article/Supplementary material, further inquiries can be directed to the corresponding author.

## Author contributions

YZ: investigation, project administration, resources, and writing – review and editing. DP: investigation, data curation, and writing – review and editing. PX: investigation, formal analysis, and writing-review and editing. QX: investigation, and writing – review and editing. FG: formal analysis, and writing – review and editing. XZ: investigation, writing – review and editing, supervision, investigation, formal analysis, and writing – original draft. HX: supervision and investigation. All authors contributed to the article and approved the submitted version.

## Funding

This work was supported by the National Natural Science Foundation of China (No. 32100065), the Natural Science Foundation of Shandong Province of China (No. ZR2018QC002), and the Doctoral Research Startup Foundation of Liaocheng University (No. 318052041).

## Conflict of interest

The authors declare that the research was conducted in the absence of any commercial or financial relationships that could be construed as a potential conflict of interest.

## Publisher’s note

All claims expressed in this article are solely those of the authors and do not necessarily represent those of their affiliated organizations, or those of the publisher, the editors and the reviewers. Any product that may be evaluated in this article, or claim that may be made by its manufacturer, is not guaranteed or endorsed by the publisher.

## Footnotes

^1^http://drive5.com/uparse/

^2^http://rdp.cme.msu.edu/
