# Supplementary material for: A novel lytic polysaccharide monooxygenase from enrichment microbiota and its application for shrimp shell powder biodegradation
Source: Front Microbiol. 2023 Mar 15;14:1097492. doi: 10.3389/fmicb.2023.1097492 (PMC10057547; doi:10.3389/fmicb.2023.1097492)
Supplement: Supplementary file 1 [file Data_Sheet_1.pdf]

A novel lytic polysaccharide monooxygenase from enrichment microbiota and its application for shrimp shell powder biodegradation

Supplementary Material

Table S1 The results of carbohydrate-active enzyme gene identification using dbCAN against the CAZy database.

HMM Profile	Profile Length	Gene ID	Gene Length	E Value	Profile Start	Profile End	Gene Start	Gene End	Coverage
AA1.hmm	358	MGE363	471	1.10E-30	7	213	91	286	0.575419
AA10.hmm	178	MGE2822	526	1.20E-47	1	177	29	208	0.988764
AA12.hmm	401	MGE411	457	1.30E-20	13	399	78	452	0.962594
AA3.hmm	618	MGE1919	1173	3.30E-109	85	618	13	799	0.86246
AA3.hmm	618	MGE2502	546	1.70E-54	88	400	18	535	0.504854
AA4.hmm	522	MGE538	464	2.50E-17	50	247	24	216	0.377395
AA6.hmm	195	MGE1295	204	4.90E-85	1	195	4	201	0.994872
AA6.hmm	195	MGE1703	199	7.70E-68	2	193	4	192	0.979487
CBM20.hmm	90	MGE2239	875	4.10E-25	1	88	777	866	0.966667
CBM48.hmm	76	MGE456	728	2.30E-22	2	70	124	209	0.894737
CBM48.hmm	76	MGE457	689	9.10E-19	2	71	11	102	0.907895
CBM5.hmm	40	MGE1017	658	5.50E-17	2	40	136	174	0.95

CBM5.hmm	40	MGE1318	909	4.40E-16	2	40	130	171	0.95
CBM5.hmm	40	MGE135	439	1.40E-16	2	40	118	156	0.95
CBM5.hmm	40	MGE1931	645	2.30E-16	1	40	125	167	0.975
CBM5.hmm	40	MGE1944	570	1.10E-16	2	40	125	163	0.95
CBM5.hmm	40	MGE1945	515	1.40E-16	1	40	130	172	0.975
CBM5.hmm	40	MGE1946	545	4.70E-17	1	40	121	160	0.975
CBM5.hmm	40	MGE2091	631	6.40E-16	2	40	582	620	0.95
CBM5.hmm	40	MGE2438	525	9.30E-16	1	40	135	174	0.975
CBM5.hmm	40	MGE3078	377	1.20E-16	1	40	105	144	0.975
CBM5.hmm	40	MGE351	352	2.30E-16	3	40	97	135	0.925
CBM5.hmm	40	MGE573	667	2.70E-16	2	40	120	158	0.95
CBM73.hmm	54	MGE2789	339	2.90E-17	1	53	287	334	0.962963
CE11.hmm	271	MGE1136	304	1.30E-114	1	270	4	275	0.99262
CE2.hmm	209	MGE2403	900	5.70E-50	1	209	680	893	0.995215
CE4.hmm	130	MGE162	288	3.30E-34	5	126	62	186	0.930769
CE4.hmm	130	MGE304	291	4.90E-33	5	126	82	205	0.930769
CE9.hmm	373	MGE2652	368	6.00E-112	5	373	6	361	0.986595
GH102.hmm	157	MGE3491	398	8.50E-59	2	157	141	294	0.987261
GH103.hmm	295	MGE3046	348	5.10E-95	9	294	36	324	0.966102
GH109.hmm	399	MGE3185	345	2.30E-21	6	163	5	151	0.393484
GH109.hmm	399	MGE745	329	8.10E-18	4	173	3	163	0.423559
GH13_10.hmm	313	MGE1636	596	3.80E-129	5	313	124	425	0.984026
GH13_11.hmm	351	MGE1637	743	1.90E-163	1	351	186	535	0.997151
GH13_11.hmm	351	MGE457	689	3.30E-160	1	351	179	528	0.997151
GH13_16.hmm	353	MGE1852	548	1.10E-142	1	353	30	382	0.997167
GH13_23.hmm	344	MGE2029	533	1.50E-160	1	342	30	366	0.991279

GH13_23.hmm	344	MGE2030	526	4.70E-147	1	343	29	366	0.994186
GH13_23.hmm	344	MGE455	544	5.90E-154	1	343	30	380	0.994186
GH13_26.hmm	296	MGE1635	983	2.20E-121	2	295	73	434	0.989865
GH13_29.hmm	344	MGE1827	550	1.10E-178	1	344	25	363	0.997093
GH13_9.hmm	301	MGE456	728	2.40E-162	1	301	275	575	0.996678
GH15.hmm	361	MGE2239	875	4.90E-93	9	360	322	746	0.972299
GH16.hmm	189	MGE368	294	2.50E-20	3	186	23	252	0.968254
GH16_3.hmm	230	MGE135	439	2.00E-89	1	230	200	437	0.995652
GH18.hmm	296	MGE1318	909	6.00E-73	3	288	271	730	0.962838
GH18.hmm	296	MGE1502	357	5.60E-75	6	283	8	347	0.935811
GH18.hmm	296	MGE1543	417	7.10E-72	22	283	64	405	0.881757
GH18.hmm	296	MGE1546	760	5.20E-59	4	283	29	385	0.942568
GH18.hmm	296	MGE1901	745	2.30E-58	3	287	25	385	0.959459
GH18.hmm	296	MGE1931	645	1.20E-78	4	288	219	638	0.959459
GH18.hmm	296	MGE1941	642	3.30E-78	3	286	229	633	0.956081
GH18.hmm	296	MGE1942	648	5.90E-78	7	286	228	641	0.942568
GH18.hmm	296	MGE1943	839	7.20E-65	5	287	362	797	0.952703
GH18.hmm	296	MGE1944	570	1.50E-23	4	203	236	506	0.672297
GH18.hmm	296	MGE1946	545	1.50E-24	4	283	234	529	0.942568
GH18.hmm	296	MGE2109	477	4.90E-65	6	283	42	407	0.935811
GH18.hmm	296	MGE2403	900	9.90E-76	2	285	199	551	0.956081
GH18.hmm	296	MGE3054	401	1.90E-87	3	284	33	386	0.949324
GH18.hmm	296	MGE3055	592	4.40E-70	4	284	203	580	0.945946
GH18.hmm	296	MGE3262	408	4.40E-67	3	283	42	396	0.945946
GH18.hmm	296	MGE384	451	5.00E-24	3	204	138	388	0.679054
GH18.hmm	296	MGE57	390	1.30E-64	4	285	25	380	0.949324

GH18.hmm	296	MGE573	667	1.40E-75	4	286	223	658	0.952703
GH18.hmm	296	MGE917	571	4.90E-19	5	278	217	527	0.922297
GH19.hmm	231	MGE1017	658	2.20E-65	1	231	298	581	0.995671
GH19.hmm	231	MGE2166	591	3.00E-64	1	231	277	531	0.995671
GH19.hmm	231	MGE2194	256	4.30E-74	8	231	55	256	0.965368
GH19.hmm	231	MGE3078	377	2.50E-75	8	231	176	377	0.965368
GH20.hmm	337	MGE1836	836	2.60E-103	9	336	300	721	0.970326
GH23.hmm	135	MGE1302	635	1.30E-25	16	123	481	607	0.792593
GH23.hmm	135	MGE2171	486	6.70E-23	5	105	282	404	0.740741
GH23.hmm	135	MGE2410	528	4.90E-23	4	122	107	242	0.874074
GH23.hmm	135	MGE2874	207	1.40E-18	11	99	93	181	0.651852
GH23.hmm	135	MGE3576	465	1.20E-22	14	126	309	447	0.82963
GH24.hmm	137	MGE679	155	9.00E-41	2	137	5	147	0.985401
GH3.hmm	216	MGE2168	515	3.50E-61	6	216	65	291	0.972222
GH5_2.hmm	237	MGE2438	525	5.40E-85	2	237	239	483	0.991561
GH73.hmm	128	MGE278	298	1.90E-30	2	125	154	287	0.960938
GH8.hmm	320	MGE3582	339	3.40E-79	4	316	28	330	0.975
GH9.hmm	418	MGE3032	591	4.70E-39	5	358	110	528	0.844498
GT104.hmm	379	MGE2053	377	4.20E-124	4	374	3	374	0.976253
GT105.hmm	131	MGE1088	625	3.90E-23	6	124	84	222	0.900763
GT19.hmm	354	MGE2250	392	2.90E-131	1	353	15	373	0.99435
GT2.hmm	170	MGE1001	345	7.90E-36	1	168	9	173	0.982353
GT2.hmm	170	MGE1089	285	1.40E-30	1	127	4	122	0.741176
GT2.hmm	170	MGE1169	259	1.00E-32	1	120	7	124	0.7
GT2.hmm	197	MGE1250	395	5.20E-24	3	192	138	334	0.959391
GT2.hmm	170	MGE2045	352	3.00E-17	1	114	31	140	0.664706

GT2.hmm	170	MGE2973	317	3.40E-18	2	165	10	164	0.958824
GT2.hmm	170	MGE3580	758	8.90E-42	1	170	138	328	0.994118
GT2.hmm	197	MGE3580	758	8.10E-24	2	187	237	434	0.939086
GT2.hmm	170	MGE560	244	1.80E-19	1	126	4	125	0.735294
GT2.hmm	170	MGE903	1476	7.00E-24	1	168	10	178	0.982353
GT2.hmm	170	MGE903	1476	2.70E-25	1	130	315	438	0.758824
GT2.hmm	170	MGE903	1476	4.00E-27	1	169	859	1039	0.988235
GT2.hmm	170	MGE997	326	2.90E-38	1	168	6	169	0.982353
GT25.hmm	181	MGE1149	252	4.30E-34	3	178	2	179	0.966851
GT26.hmm	171	MGE2049	252	8.40E-68	2	171	56	225	0.988304
GT28.hmm	157	MGE1130	358	3.10E-50	2	157	187	345	0.987261
GT30.hmm	177	MGE3169	418	1.30E-70	2	175	35	210	0.977401
GT35.hmm	674	MGE461	815	5.00E-283	1	674	101	811	0.998516
GT4.hmm	160	MGE1091	405	1.80E-32	12	157	219	370	0.90625
GT4.hmm	160	MGE1099	403	1.60E-20	9	157	217	362	0.925
GT4.hmm	160	MGE1143	375	3.40E-26	6	152	200	340	0.9125
GT4.hmm	160	MGE1144	372	9.80E-37	7	159	189	340	0.95
GT4.hmm	160	MGE1865	441	1.10E-39	5	159	216	380	0.9625
GT4.hmm	160	MGE2047	353	3.60E-31	11	152	176	316	0.88125
GT4.hmm	160	MGE81	349	5.00E-22	5	129	175	284	0.775
GT5.hmm	472	MGE1638	493	6.70E-156	2	471	5	478	0.993644
GT5.hmm	472	MGE460	475	1.90E-170	1	471	2	470	0.995763
GT51.hmm	177	MGE1694	767	3.70E-69	2	177	57	232	0.988701
GT51.hmm	177	MGE1759	236	4.60E-60	12	176	66	230	0.926554
GT51.hmm	177	MGE78	774	5.20E-48	12	176	55	218	0.926554
GT59.hmm	404	MGE1753	403	1.40E-21	8	169	32	176	0.398515

GT59.hmm	404	MGE1753	403	1.90E-16	216	404	189	368	0.465347
GT83.hmm	540	MGE271	540	2.10E-29	8	435	18	451	0.790741
GT9.hmm	225	MGE1142	364	2.50E-55	2	217	83	335	0.955556
GT9.hmm	225	MGE1145	375	2.00E-41	4	210	95	319	0.915556
GT9.hmm	225	MGE1148	351	7.70E-37	3	203	85	324	0.888889
GT9.hmm	225	MGE1863	394	2.00E-52	3	222	84	334	0.973333
GT9.hmm	225	MGE1868	341	8.30E-45	3	204	99	323	0.893333
GT9.hmm	225	MGE1869	373	3.30E-43	3	216	98	335	0.946667
GT9.hmm	225	MGE2164	337	3.90E-60	1	222	70	316	0.982222
GT9.hmm	225	MGE3170	307	4.00E-42	2	194	65	277	0.853333
GT9.hmm	225	MGE3460	366	1.30E-45	4	215	87	321	0.937778
PL12_3.hmm	144	MGE2421	567	1.20E-26	12	135	348	470	0.854167
PL12_3.hmm	144	MGE2428	689	2.70E-32	1	144	452	595	0.993056
PL14_3.hmm	190	MGE2762	637	3.70E-63	2	189	357	543	0.984211
PL15.hmm	134	MGE1441	817	6.40E-28	8	134	548	691	0.940299
PL15.hmm	134	MGE2048	554	1.40E-23	7	132	307	431	0.932836
PL15.hmm	134	MGE595	843	2.70E-24	8	132	562	711	0.925373

Table S2. Nucleic acid and amino acid sequence of M2822

	DNA sequence (5'→3')
M2822	ATGAAGTCGACGAGATCCTTTGCGGTGAAGCCGCTGAAGCTCGCCCTGACCGTCGCGCTCGCCCTGCCGGCCAGCGTCTTCGGGCACGG<br>CTCGATGGAAGTGCCGGTAAGCCGCGTGCTCAACTGTTTCAACGAAGGTCCCGAAGCGCCCAAGTCGGCCGCCTGCCAGGCCGCAGTC<br>GCCGTTTCCGGCCCGCAGATGCTGTACGACTGGTCCGGGGTCAACCAGAACCCCAATGGCAACCACCAGGCTTTCGTGCCGGACGGCCA<br>GCTGTGCGCCGGCGGCAAGTCCAACTACGCCGGCCTCGATCTGCCGCGCCAGGACTGGGTGACCACGCCGATCGCCCCCAACTCCAAC<br>GGCAACTTCGAGTTCATCTACTACGCGCCCGCCCAGCACGCCACCAGGAACTGGGTGTTCTACGTGACCAAGGATGGCTGGGATCAGA<br>GCCGCCCGCTGCGCTGGTCCGACCTGGAACAGTTCTGCACGCTCGGCAACGTGCCCGGCGACGCCAACAAGCGCTACCACCTGACCTGC<br>CCGCTGCCCAAGAACAAGACCGGCAAGCACATCATCTTCAACACCTGGCAGCGCTCGGACAGCCCTGAAGCGTTCTACTCGTGCTCGGA<br>CGTGAGCTTCTCGCCCATCGTCTCCAACTACAAGGAACTGGGCCAGATCCGTTCCTCGCAGGATCTGAAGGACGAAACCAAGGTCACCT<br>TCCGGCTGTTCGACGCCAACGGCGCCGACCTGGAAACCTTCACCGTGGTGATGACCTACAACCCGGCCAACGGCGACCAGATGTTCCTG<br>GCCAACCTGTGGCCGTACTACCTTGCCCAGAACGTGAACGCGGCGTCGCGCTACGTCAGCATCGGTGTGCTCTCCACCAACGGCAGCGT<br>CTCGCCGGCCAAGAGCACCCAGGACAACCGCGTGTACTCGCGTGACGGTGGCAACTACACCTTCCGTGTCGACATCGCCGGCGTCGTCG<br>CGCCGACCCCGACGCCGGTCACCCCGACCCCGACGCCGGTGACCCCCACGCCGAACCCGGTCACCCCGACCCCCACCCCGGTCACGCC<br>GACCCCGACGCCGGTGACCCCCACGCCGACGCCGGTCACCCCGACGCCGGCCCCGGGCACCTGCCACGCCGCCTGGGCTGAAGGCAAG<br>ACCTACAACGCCGGCACGCTGGTCACCTACAATGGCCGCAACTACCAGGCCCTGGTGACGCACGTGGCCCACGTGGGCGCCAACTGGA<br>ACCCGGCCTCCAGCAACACGCTGTGGAAGGATGTCGGCGCCTGCACCGGCACCGCCACTCCGACGCCGGCCACGCCGACACCGACCCC

	GGTCACCCCGACCCCGACGCCGGTGACCCCGACGCCGAGCCCGGTGACGCCGACGCCGGCCACTCCGACGCCGACGCCTACCCCGGGC<br>ACCGGCTGCGCGCCGGCCTGGTCCGCCAGCACCGTCTACGCCGCCTCCAACACCAAGGTGAGCTACAACGGCCGCAACTACCAGAACA<br>AGTGGTGGACCCAGGGCGACAACCCGTCCCAGACCGGTCAGTGGGGCGTCTGGCAGGATCTGGGTACCTGCCAGTAA
	Amino acid sequence
	MKSTRSFAVKPLKLALTVALALPASVFGHGSMEVPVSRVLNCFNEGPEAPKSAACQAAVAVSGPQMLYDWSGVNQNPNGNHQAFVPDGQL<br>CAGGKSNYAGLDLPRQDWVTTPIAPNSNGNFEFIYYAPAQHATRNWVFYVTKDGWDQSRPLRWSDLEQFCTLGNVPGDANKRYHLTCPLP<br>KNKTGKHIIFNTWQRSDSPEAFYSCSDVSFSPIVSNYKELGQIRSSQDLKDETKVTFRLFDANGADLETFTVVMTYNPANGDQMFLANLWPYY<br>LAQNVNAASRYVSIGVLSTNGSVSPAKSTQDNRVYSRDGGNYTFRVDIAGVVAPTPTPVTPTPTPVTPTPNPVTPTPTPVTPTPTPVTPTPTPVT<br>PTPAPGTCHAAWAEGKTYNAGTLVTYNGRNYQALVTHVAHVGANWNPASSNTLWKDVGACTGTATPTPATPTPTPVTPTPTPVTPTPSPVT<br>PTPATPTPTPTPGTGCAPAWSASTVYAASNTKVSYNGRNYQNKWWTQGDNPSQTGQWGVWQDLGTCQ
